# High-Density, Nonvolatile, Flexible Multilevel Organic Memristor Using Multilayered Polymer Semiconductors

**DOI:** 10.1021/acsami.4c03111

**Published:** 2024-04-22

**Authors:** Shubham Sharma, Manish Pandey, Shuichi Nagamatsu, Hirofumi Tanaka, Kazuto Takashima, Masakazu Nakamura, Shyam S. Pandey

**Affiliations:** † Graduate School of Life Science and Systems Engineering, Kyushu Institute of Technology, 2-4 Hibikino, Wakamatsu, Kitakyushu 808-0196, Japan; ‡ Department of Electronics and Communication Engineering, Indian Institute of Technology, Durg,Bhilai, Chattisgarh 491001, India; § Department of Computer Science and Electronics, Kyushu Institute of Technology, 680-4 Kawazu, Iizuka 820-8502, Japan; ∥ Department of Human Intelligence Systems, Kyushu Institute of Technology, 2-4 Hibikino, Wakamatsu, Kitakyushu 808-0196, Japan; ⊥ Division of Materials Science, Nara Institute of Science and Technology, Ikoma, Nara 630-0192, Japan

**Keywords:** semiconducting polymers, resistive memory devices, floating film transfer method, multilevel switching, flexible devices

## Abstract

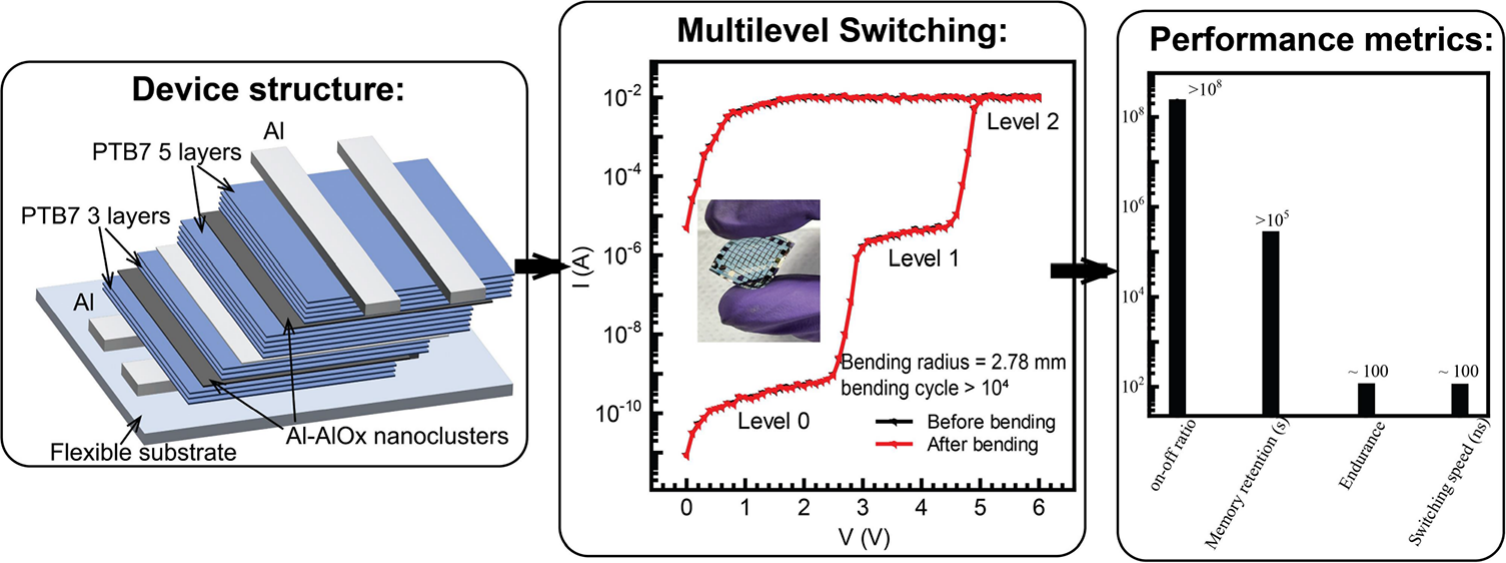

Nonvolatile organic memristors have emerged as promising candidates for next-generation electronics, emphasizing the need for vertical device fabrication to attain a high density. Herein, we present a comprehensive investigation of high-performance organic memristors, fabricated in crossbar architecture with PTB7/Al-AlO_*x*_-nanocluster/PTB7 embedded between Al electrodes. PTB7 films were fabricated using the Unidirectional Floating Film Transfer Method, enabling independent uniform film fabrication in the Layer-by-Layer (LbL) configuration without disturbing underlying films. We examined the charge transport mechanism of our memristors using the Hubbard model highlighting the role of Al-AlO_*x*_-nanoclusters in switching-on the devices, due to the accumulation of bipolarons in the semiconducting layer. By varying the number of LbL films in the device architecture, the resistance of resistive states was systematically altered, enabling the fabrication of novel multilevel memristors. These multilevel devices exhibited excellent performance metrics, including enhanced memory density, high on–off ratio (>10^8^), remarkable memory retention (>10^5^ s), high endurance (87 on–off cycles), and rapid switching (∼100 ns). Furthermore, flexible memristors were fabricated, demonstrating consistent performance even under bending conditions, with a radius of 2.78 mm for >10^4^ bending cycles. This study not only demonstrates the fundamental understanding of charge transport in organic memristors but also introduces novel device architectures with significant implications for high-density flexible applications.

## 1 Introduction

There has been a growing interest in organic resistive memristors in recent years^1−4^ due to the constraints emerging from the downscaling limits of conventional silicon-based technologies.^5−7^ Conventional devices encounter challenges related to energy consumption, affordability, and flexibility within the framework of the traditional Von-Neumann architecture.^8^ In comparison, organic devices have garnered significant attention, encompassing from the cost-effectiveness and flexibility to the lightweight and simple device architecture enabling significant prospects for developing next-generation memory devices.^9−11^ In a typical resistive memory system, data storage relies on differences in electrical resistance induced by the applied voltage. Distinct resistive states in the recorded data can be retrieved nondestructively. The memory effect exhibits nonvolatility, implying the stability of data retention and in the absence of an applied voltage. In general, organic memristors operate as binary systems, characterized by two distinct conductive states: the 'ON' and 'OFF' states.^12,13^ However, employing a multilevel memory strategy for high-density data storage [≥3^*n*^; where *n* = number of bits in a device] emerges as a proficient alternative,^14−16^ enabling the capability to store more than two distinct states within the same device. These devices are energy efficient and offer a faster access time by accessing multiple states simultaneously. Literature shows the different mechanisms, encompassing redox-driven switching,^17,18^ filament formation processes,^19,20^ and the utilization of optically responsive materials^21−23^ for charge storage. These storage techniques have challenges related to material degradation over time (in redox-driven switching), inconsistent and unpredictable storage (in filament-driven switching), and the complexity and availability of the photosensitive material (in optically responsive switching). Recently, perovskite-based memristors also geared attention due to their high performance and fast switching, but possess challenges like stability of perovskite materials and lead (Pb)-related toxicity. While Pb-free perovskite-based memristors offer excellent performance, the processing and characterization of these materials necessitate the use of an inert atmosphere.^24^ Further, the flexibility of such devices has always remained a major concern for inorganic as well as inorganic–organic hybrid semiconductors. On the contrary, semiconducting polymers (SCPs) are the most stretchable semiconductors and are exceptionally well-suited for the fabrication of large-area printable electronic devices.^25,26^

Among different organic memristor architectures, the crossbar emerges as a prominent choice due to its simplicity and effectiveness in enabling vertical stacking for scalability. Crossbar architecture with arrays of top and bottom electrodes separated by thin semiconducting layers enables high memory density. In one of the cross-bar memristors, a thin layer of metal/metal-oxide nanoparticles can be used to facilitate the modulation of molecular resistance through the applied bias; however, introducing such layers between the SCP layers is challenging due to the limited choices for the selection of suitable orthogonal solvents.^27^ Therefore, an alternative approach involves to incorporate metal/metal-oxide nanoparticles into SCP through the preparation of their composites.^28,29^ Layer-by-Layer (LbL) deposition of SCP films using solution processing is although challenging but plays a pivotal role toward the fabrication of three-dimensional integrated organic memristors. Therefore, conventional solution processable techniques like spin coating, spray coating, doctor blading, etc. are not suitable. Although LbL film of small organic molecules can be fabricated using vacuum evaporation, high fabrication cost, scalability, and low flexibility of the film still remain a major challenge, which needs their amicable solutions.

**Figure 1:**
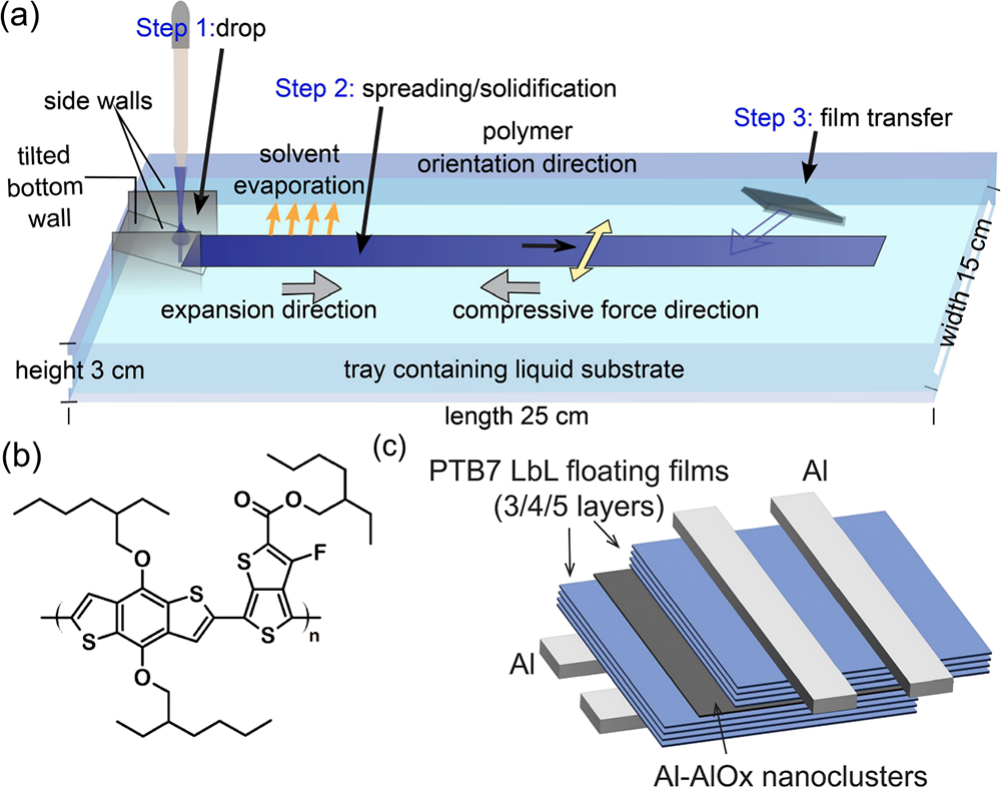
(a) Schematic representation of the UFTM. (b) Chemical structure of PTB7. (c) Device schematic of organic memristors. The PTB7 floating films deposited in LbL with 3, 4, and 5 layers with thickness 61.7, 82.4, and 104.3 nm, respectively.

There has been significant interest in the fabrication of SCP films on a liquid substrate, which can subsequently be transferred onto the device substrate after solidification.^30^ One such method, known as Unidirectional Floating-Film Transfer Method (UFTM) developed by our group is an excellent film fabrication method for the deposition of large-area thin films of SCP (Figure 1a, Supporting Video 1).^31−33^ A drop (∼10 μL) of SCP solution is dropped onto the hydrophilic liquid substrate, facilitating controlled spreading; as the solvent gradually evaporates, resulting in a solid floating thin film. The efficacy of this method lies in the independent control on film formation and their transfer on any desired substrate enabling facile LbL coating without hampering the underlying layers.^34^

In this work, we have demonstrated the fabrication of an organic memristor using LbL deposited poly[[4,8-bis[(2-ethylhexyl)oxy]benzo[1,2-b:4,5-b’]dithiophene-2,6-diyl][3-fluoro-2-[(2-ethylhexyl)carbonyl]thieno[3,4-*b*]thiophenediyl]] (PTB7) (chemical structure shown in Figure 1b) floating films. This excellent crossbar device architecture with PTB7/Aluminum–Aluminum oxide (Al-AlO_*x*_) nanocluster/PTB7 between Al contacts (Figure 1c), offers a compelling synergy of facile fabrication, cost-effectiveness, and meticulous control over resistive states. The thickness of the two SCP films shown in the device structure was adjusted by varying the number of layers through LbL deposition. Subsequently, their impact on the current–voltage (*I*–*V*) characteristics revealed that an increase in the thickness of the semiconducting layers corresponds to an increase in the resistance of the resistive states. Devices subjected to different LbL deposition demonstrated exceptional performance metrics, including a high on–off ratio (>10^5^), extended retention time (>10^5^ s), high endurance (∼100 cycles), and rapid switching (∼100 ns) into different states. We fabricated 64 devices on a 2 cm × 2 cm substrate in the crossbar architecture, achieving a success rate of over 95% for the operational devices. By employing the Hubbard model to unravel the charge transport mechanism, we highlighted the pivotal role played by Al-AlO_*x*_ nanoclusters in the process. The switching-on of our devices was attributed to the accumulation of bipolarons in the SCP layer, contributing significantly to the understanding of organic memristors in such a crossbar structure with Al-AlO_*x*_ nanoclusters. Pushing beyond traditional architectures, we propose a vertically stacked configuration for multilevel switching, effectively doubling device density. A device architecture with vertically stacked 3- and 5-layer configurations, separated by a thin dielectric, demonstrates three distinct states, achieving an outstanding on–off ratio (>10^8^), high memory retention (>10^5^ s), robust endurance (87 cycles), and rapid switching (∼100 ns) between on- and off-states. We further demonstrated the applicability of these device architectures and fabrication techniques on flexible substrates, revealing that flexible devices can maintain similar performance even after being bent at a radius of 2.78 mm for 10^4^ cycles.

## 2 Results and Discussion

### 2.1 Thin Film Microstructure

**Figure 2:**
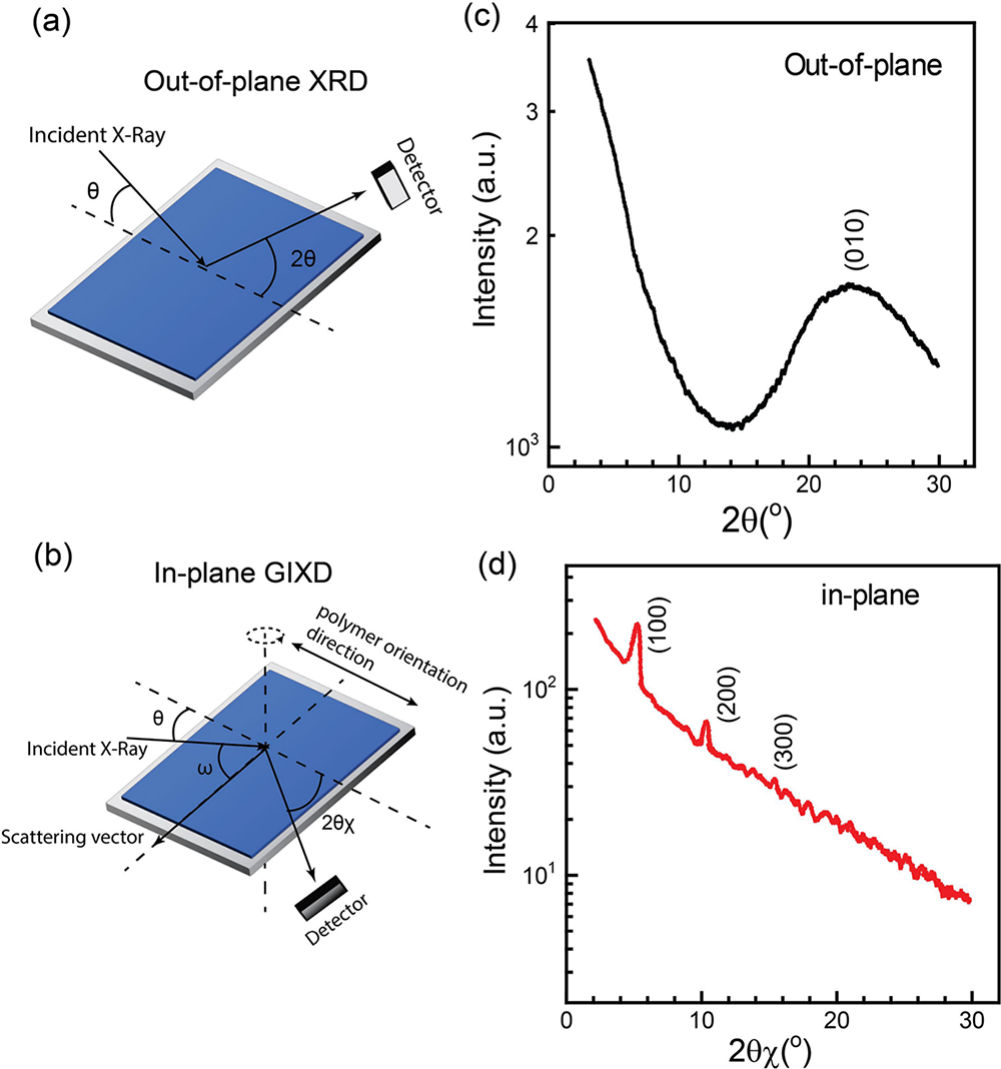
Microstructural Characterization of PTB7 floating films by XRD: Sketch of X-ray measurement used (a) out-of-plane XRD (b) in-plane GIXD. XRD patterns of PTB7 films: (c) out-of-plane, (d) in-plane.

Film microstructures of PTB7 films were analyzed using out-of-plane X-ray diffraction (XRD) and in-plane grazing incidence XRD (GIXD) in the geometry described in Figure 2a,b. The out-of-plane XRD (Figure 2c) revealed a (0*k*0) diffraction peak at 2θ = 23.6°, indicative of π–π stacking. In contrast, the in-plane GIXD (Figure 2d) displayed a series of (*h*00) diffraction peaks corresponding to alkyl chain stacking up to third order at 2θχ = 5.1, 10.2, and 15.4°. The combination of out-of-plane and in-plane diffraction results shows that the PTB7 is mainly face-on oriented with π–π and alkyl chain stacking horizontal to the substrate surface enabling facile charge carrier transport in the vertical direction.^35,36^ During the fabrication of films on a hydrophilic liquid substrate in UFTM, the SCP backbone with hydrophilic side chains tends to form a face-on-dominated conformation. PTB7, incorporating hydrophilic moieties within its alkyl side chains, exhibits a predominant face-on population of polymeric domains utilizing UFTM. Note that SCPs adopting a face-on orientation are widely recognized for their efficiency in promoting charge transport in the vertical direction, as also observed in applications like organic photovoltaics and light-emitting diodes.

### 2.2 Device Characterization

#### 2.2.1 Bistable Switching

**Figure 3:**
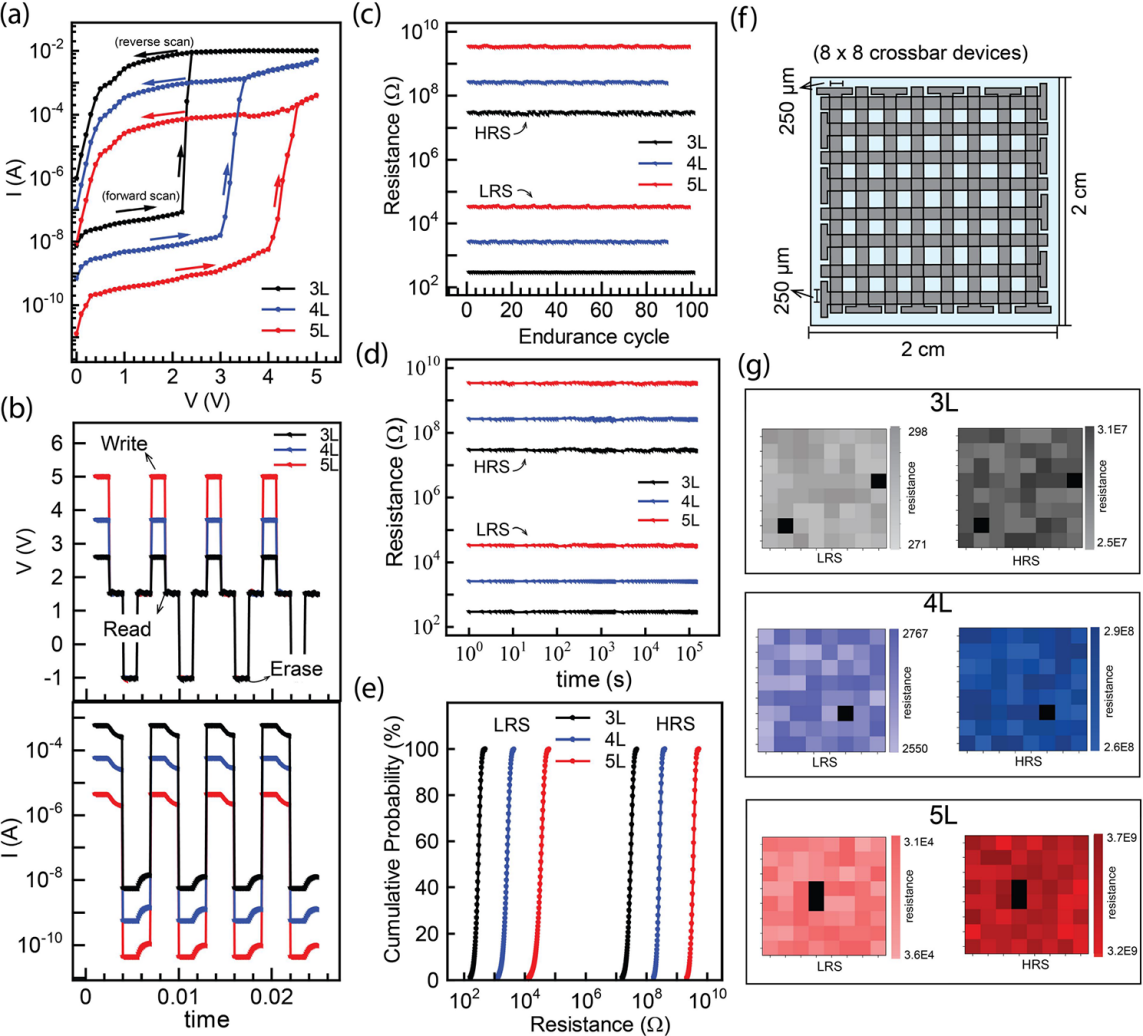
Characteristics for Bistable Switching: (a) *I–V* characteristics, (b) AC response showing *V–t* and its corresponding *I–t* characteristics, (c) endurance cyclability of LRS and HRS, (d) retention time of LRS and HRS, (e) cumulative probability data of LRS and HRS for 3 (black), 4 (blue), and 5 (red) layer configurations of organic memristors, (f) schematic of our high-density crossbar architecture, and (g) device-to-device uniformity for 3-, 4-, and 5-layer configurations of the devices. Black marked devices are nonworking devices.

As shown in Figure 1c, the device comprises a bottom Al electrode, followed by 3, 4, and 5 layers of PTB7 films fabricated by LbL. Notably, the film thickness of each UFTM layer in all devices was uniform and consistent (Supplementary Figure S1). Subsequently, a thin layer (∼10 nm) of Al-AlO_*x*_ nanoislands is thermally evaporated under a high vacuum (10^–6^ Torr) at a slow deposition rate (<0.1 Å s^1–^). The residual oxygen inside the evaporator results in the formation of distinct layers comprising AlO_*x*_ nanoislands interspersed within layers of Al nanoparticles. It was succeeded by another top 3, 4, and 5 layers of PTB7 films fabricated by LbL. Finally, the top Al electrode was deposited in crossbar architecture to complete the device. The *I*–*V* characteristics of organic memristors thus fabricated are shown in Figure 3a. The *I–V* characteristics were measured from 0 to 5 V in a forward scan and then 5 to 0 V in a reverse scan. In the forward scan, at 0 V, the devices are in the off-state, with a low current of 4.1 × 10^–8^, 4.7 × 10^–9^, and 3.5 × 10^–10^ A for 3, 4, and 5-layer configurations, respectively. An increase in the applied voltage results in a small increase in the current from 0 to 2.3, 3.5, and 4.6 V for 3, 4, and five layers of LBL-coated PTB7, respectively). The initial phase, when the system is in a quiescent state, exhibiting low conductance, is also termed a high resistance state (HRS). However, upon surpassing threshold critical voltage (*V*_C_), a substantial increase in the injection current can be observed (4.8 × 10^–3^, 5.5 × 10^–4^, and 3.9 × 10^–5^ A for 3, 4, and 5-layer configuration, respectively), indicating the onset of enhanced conductance and are in the low resistance state (LRS). The on–off ratio for each device configuration was found to be >10^5^. As the semiconductor film thickness increases, device resistance concurrently rises, resulting in higher resistance of the LRS and HRS. This emphasizes the influence of film thickness on electrical characteristics, providing insights that bear significance for the design and optimization of SCP-based electronic devices. Further, it can be observed that *V*_C_ also increases with an increase in the thickness of the SCP films (Supplementary Figure S2). The values for *V*_C_ were 2.3 3.5, and 4.6 V for 3, 4, and 5-layer devices. The electrical field in the devices is typically concentrated near the Al-nanocluster/organic interface. A thicker organic layer distributes the electric field over a larger dimension, resulting in higher voltage required to initiate the switching process. These distinctive characteristics observed in the *I–V* curve of the organic memristors with varying thicknesses exhibit a unique attribute. These characteristics also signify the feasibility of implementing multilevel switching within the same device. Here, it is worth noting that *V*_C_ can be precisely controlled by simply adjusting the number of layers during the device fabrication (Figure S2, Supporting Information). Consequently, *V*_C_ can be predicted and tuned for utilization in multilevel switching in memristors. The fabrication of devices demonstrating multilevel switching will be explained later. Once the devices are switched on, they tend to remain in the LRS even when the voltage is decreased in the reverse scan (5–0 V). The devices maintain their on-state, after the removal of the voltage supply, exhibiting the nonvolatile behavior.

Next, we examined the AC characteristics of the three memristors, consisting of 3, 4, and 5 layers of LBL-coated PTB7, and the results are presented in Figure 3b. The transition from HRS to LRS corresponds to the “writing” process in a digital memory device. Each of the three organic memristors was subjected to voltages marginally exceeding their respective threshold *V*_C_ to initiate the writing process and was subsequently read at 1.5 V. The *I–t* characteristics at *V*_read_ indicate that the devices remain in the on-state. Later, at −1 V, a succeeding transition from on to off-state is observed. This process is known as “erasing” or “reset” process. Upon subsequently reading the devices, off-state or HRS was observed, indicating that the devices were erased. Importantly, the states of the devices persisted even after bias removal, indicating a nonvolatile memory effect. Moreover, this process could be repeated multiple times, affirming the reversibility of the memristors. We quantified the switching speed of the devices by assessing their AC characteristics (Figure S3, Supporting Information). Our analysis revealed a fast switching speed of ∼100 ns, demonstrating the devices’ impressive capability to transition between on and off states. This rapid switching speed signifies the practical feasibility of swiftly toggling the devices between distinct operational states. Consequently, on and off endurance cycles were systematically measured to test the stability of the devices, as shown in Figure 3c. The devices exhibited remarkable stability, enduring 101, 92, and 98 cycles for 3-, 4-, and 5-layer configurations, respectively, with <1% change observed throughout the cycling process. Upon investigating the long-term retention times of the HRS and LRS (Figure 3d), we observed no obvious decline over an extended duration of 2 × 10^5^ s. According to the Rayleigh instability model, the retention time in each state is intricately linked to the surface energy of the top and bottom contacts and the activation energy required for the injection of charge carriers while switching. A higher energy barrier prolongs the retention time but requires a higher voltage for switching. In this trade-off between retention time and *V*_C_ for switching, our organic memristors exhibited optimal performance at both ends. Given the random nature of the SCPs, the device-to-device variation of organic memristors remains a point of concern. We fabricated 64 devices on a 2 cm × 2 cm substrate with each device area of 6.25 × 10^–2^ mm^2^ as shown in Figure 3f. Figure 3e presents the distribution of the resistance for the 64 devices for 3/4/5-layer configurations. The mean values for LRS were 292 ± 16.1, 2601 ± 131, and 3.4 × 10^4^ ± 1.7 × 10^3^ Ω for 3, 4, and 5-layer device configurations, respectively. The mean values for HRS were 3.1 × 10^7^ ± 2.2 × 10^6^, 2.63 × 10^8^ ± 8.5 × 10^6^, and 3.73 × 10^9^ ± 7.8 × 10^7^ Ω for 3-, 4-, and 5-layer device configurations, respectively. The distribution of *V*_C_ for the 64 devices on a single substrate is given in Figure S4, Supporting Information. The high uniformity of the devices and the capability of fabricating high-density devices in a small area, with minimal cross-talk, are also demonstrated, as shown in Figure 3g. Notably, >95% of devices in each of the three configurations exhibited uniform functionality. The *I–V* characteristics of all the devices are shown in Figure S5, Supporting Information. The low crosstalk stems from the substantial 1 mm lateral separation between neighboring devices compared to their 60–100 nm vertical thickness. This architectural design, with ultrathin vertical profiles and large lateral spacing, enables high-density integration while minimizing electric field interference and crosstalk between neighboring cells. Even with increased device density in the same area, crosstalk can be kept under control as long as the lateral separation between neighboring cells remains significantly larger than the vertical thickness. Additionally, device-to-device variation for 30 different batches is presented in Figure S6, Supporting Figure. Here, we considered the average on–off ratio for 64 devices in each batch. There is a variation of less than 5% in the different batches. The consistent performance observed in the devices can be ascribed to the excellent uniformity of the films prepared by UFTM, making a substantial contribution to the overall reliability and stability.

#### 2.2.2 Conduction Mechanism

**Figure 4:**
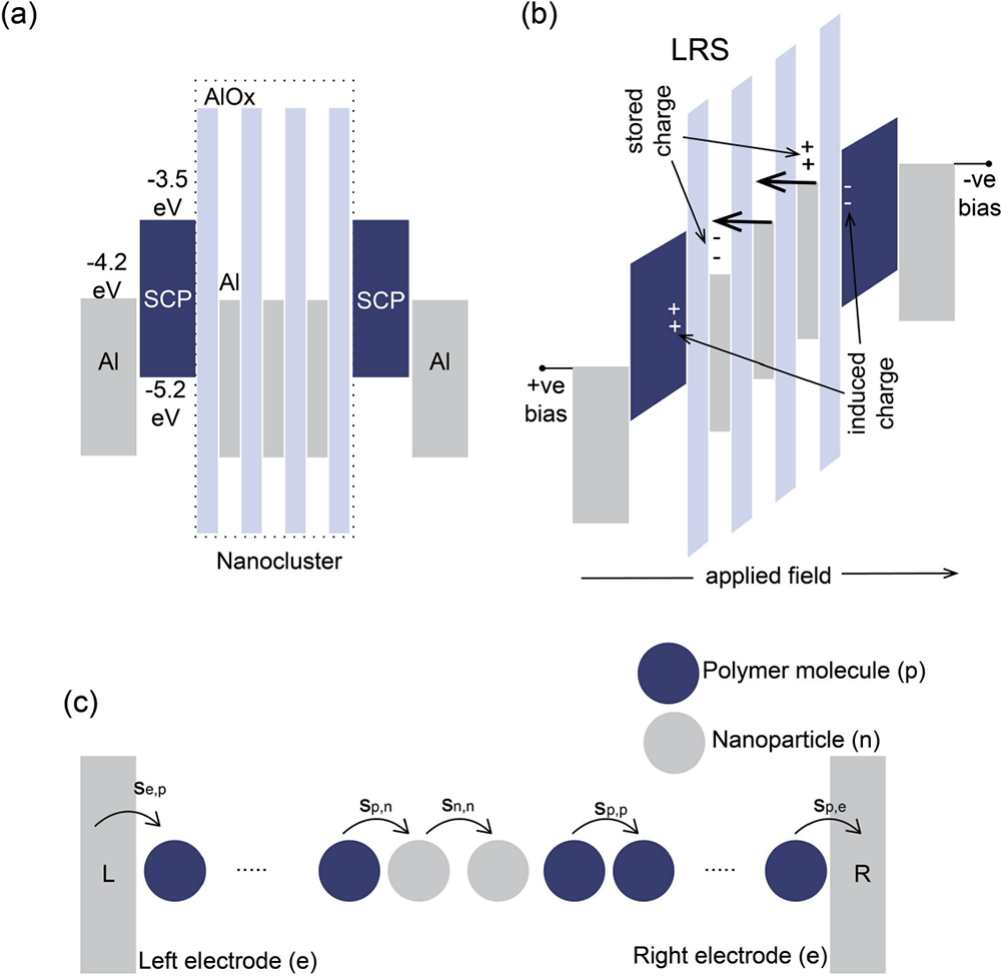
Conduction mechanism: (a) schematic showing the energy diagram of the memristors, (b) effect of applied bias, and (c) structure of our memristors showing the hopping mechanism.

To understand the dynamics of our organic memristors, a fundamental aspect is to comprehend the charge transport mechanism within the device structure. Numerous device frameworks and mechanisms have been proposed to unravel the memory effects of the devices,^37,38^ which include the transformation of the macromolecular domains from amorphous to extended-ordering,^39,40^ formation of conduction channel between the two contacts,^41,42^ intramolecular rotation of the molecular complexes,^43,44^ and by coinciding the tails of valence and conduction bands.^45^ Nevertheless, the efficacy of our memristors is dependent on and influenced by the Al-AlO_*x*_ nanoclusters. The Al-AlO_*x*_ nanoclusters deposited in the thermal evaporator at a gradual rate (∼0.1 Å s^–1^) result in the formation of a sequence of energy wells of Al and AlO_*x*_, as illustrated in Figure 4a. The changes in the device resistance were attributed to the redistribution of charges within the nanocluster layer. In this section, we thoroughly investigated the charge transport mechanism of our memristors. Previous studies demonstrated that the charge transport across the nanoclusters forms distinct positive–negative electronic regions.^36,38^ To explain this phenomenon in the proposed memristor under investigation, we assume that the Al/PTB7/Al-AlO_*x*_ nanocluster/PTB7/Al configuration is a 1-D structure. Considering SCP molecules and nanoparticles as *p* and *n*, respectively, and the total number of particles as T in the device configuration, we aim to understand the interaction between two SCP molecules and the interaction between an SCP molecule and a nanoparticle. Let us denote two SCP molecules as *p*_o1_ and *p*_o2_, such that *T* = *p*_o1_ + *p*_o2_ + *n*. The Hubbard model will be used to investigate the interaction of SCP molecules and nanoparticles.^46^ Every site in the device structure signifies either an SCP molecule or a nanoparticle. The interaction between two adjacent sites, denoted as *s*_*ij*_, is dictated by the hopping term (Figure 4c). A weak interaction between the adjacent sites is assumed to be significantly less than the local Coulomb interaction *U*(*i*). Strong *U*(*i*) in the electrons induces the energy bands to split into upper and lower sub-bands,^47^ distinctly separated by an energy of the order of *U_n_*. This separation of the energy band is attributed to quantum effects associated with the nanoparticles. In the absence of the applied voltage, the device remains in the non-charged state, with the lower sub-band occupied, while the upper sub-band is empty. When the applied voltage increases, the energy levels of the nanoparticles near the anode decrease, while those near the cathode increase due to the applied electric field. As a result, the energy levels of the nanoparticles at the lower sub-band near the cathode align with the energy levels of the nanoparticles at the upper sub-band near the anode. This enables the resonant tunneling of electrons from the lower sub-band on one side of the nanocluster layer to the upper sub-band on the other side, creating partially occupied states. These states are responsible for switching the devices, as illustrated in Figure 4b. This behavior is reminiscent of the treatment of hopping in the metallic granular medium.^48^ The applied bias at which the electrons can tunnel through neighboring nanoparticles is *V*_C_. With a continued increase in bias, the system remains in a charged or on-state. Upon decreasing the applied bias, the trapped charges persist due to the high barrier between the two nanoparticles arising from the AlO_*x*_ insulation. This behavior underscores the nonvolatile memory characteristics of our memristors.

The Hubbard model initially formulated to elucidate band magnetism in transition metals, has evolved into a fundamental framework for investigating the intrinsic physics of strongly correlated electron systems over the years.^49,50^ While the Hubbard model is conceptually simple, it poses a nontrivial many-body problem.^51^ However, we have used an approximation to understand the charge transport mechanism, for which we have taken a 1-D system comprising two electrodes, an SCP, and a nanocluster (metal and metal oxide nanoparticles), as shown in Figure 4c. The redistribution of the carriers in the nanocluster serves as the origin of the memory effect. The Hamiltonian of the device configuration can be expressed by eq 1(46)
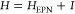
1Where two Hamiltonians are defined, *H*_EPN_ for the noninteracting molecules, (left (L), right (R) electrodes, SCP molecules, and nanoparticles), and other *I* for the interaction between electrodes and SCP molecules. The non-interacting Hamiltonian can be written as
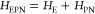
2where *H*_E_ and *H*_PN_ are the Hamiltonians of the two electrodes and nanoparticles, respectively.
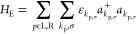
3Here, *a*_*k*_p,σ__^+^ (*a*_*k*_p,σ__) are creation (destruction) operators for an electron of energy ε_*k*_p,σ_,_ and spin σ. The wave vector of the electron is represented by *k*_p_. The Hamiltonian includes the kinetic energy associated and captures the electronic structure when a bias is applied to the device. The creation (annihilation) operator signifies the addition (removal) of the electron to the electronic state. The Hamiltonian for understanding the SCP molecules and nanoparticles is
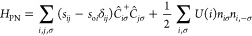
4where *Ĉ*_*i*σ_^+^ (*Ĉ*_*j*σ_) is the creation (destruction) operator for SCP molecules and nanoparticles. *n*_*i*σ_ = *Ĉ*_*i*σ_^+^*Ĉ*_*j*σ_. *s*_*ij*_ is the hopping term between the adjacent sites. *s*_0*i*_ is the hopping term between the first site and its adjacent site.

The Hamiltonian for the interactions between SCP molecules and electrodes and nanoparticles is expressed as
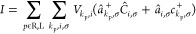
5

**Figure 5:**
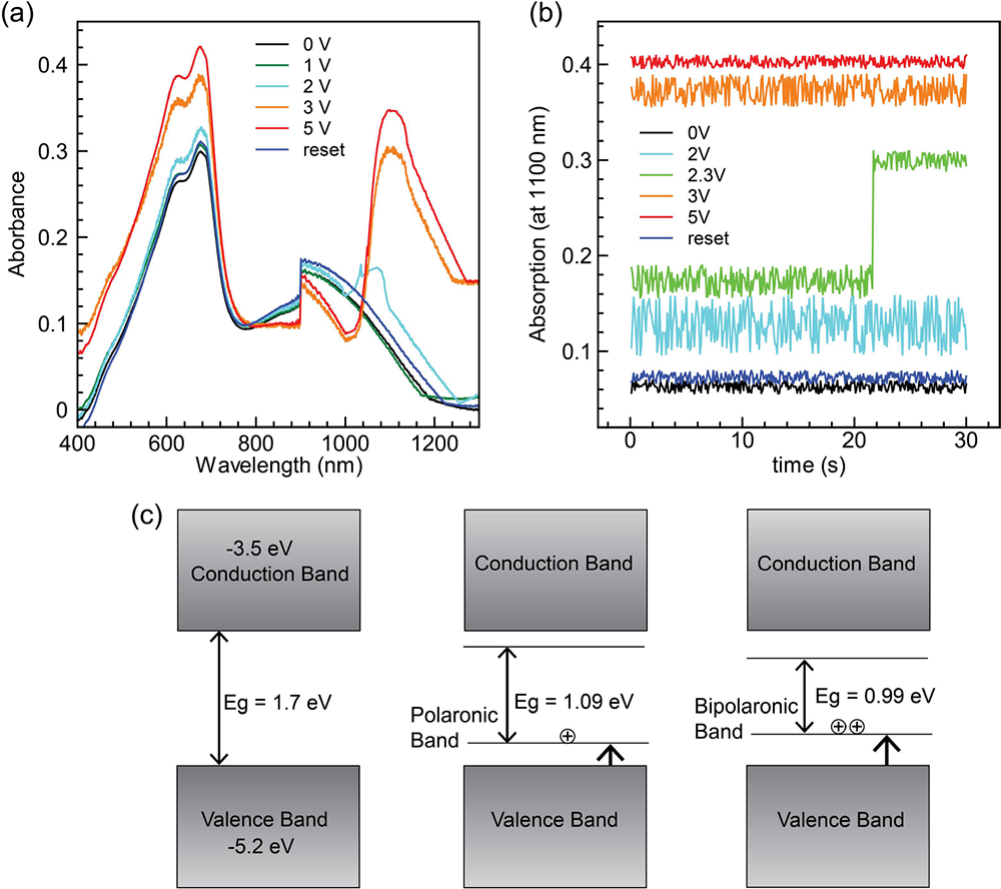
(a) Electronic absorption spectra of the memristors at a bias of 0, 1, 2, 3, 5 V, and after reset, (b) peak absorption (at 1100 nm) with respect to time, and (c) band diagram before and after the accumulation of bipolarons.

*V*_*k*_p_,*i*_ is the interaction strength between the molecules. Since we have considered a 1-D system, we suppose that the left electrode interacts only with the first SCP molecule, while the last SCP molecule interacts only with the right electrode. From eq 5, it can be understood that the transfer of one electron from the electrode/nanoparticles into the SCP sites can be represented by the destruction of the electron from the electrode/nanoparticle site and its creation into the SCP site. In the context of our 1-D system, characterized by noninteracting hopping terms, the Hubbard model elucidates the on-site charge transport phenomena within electrodes, SCP molecules, and nanoparticles. Simultaneously, interactions between electrodes/nanoparticles and SCP molecules introduce additional *s*_*ij*_. Upon applying a bias exceeding *V*_C_ to our system, the Hubbard model’s *s*_*ij*_ becomes active, facilitating the tunneling of charge carriers from the metallic core of the middle nanocluster layer. This results in the creation and destruction of electrons between adjacent nanoparticles, accompanied by the induction of charges in the SCP layers. The charges induced in the SCP layers are further influenced by their *V*_*k*_p_,*i*_ with the nanoparticle, which governs the extent of charge transfer through interfacial polarization. Consequently, the resistance of the device changes drastically through a doping mechanism, analogous to the channel formation observed in organic transistors. This pronounced doping of charge carriers into the SCP film at voltages >*V*_C_ results in a sharp increase in current. Notably, polarons emerge as the predominant charge-carrying entities in the SCP, typically delocalized over a small SCP segment (2–4 monomer units). They encompass an electron or hole within the intramolecular framework and further incorporate electronic polarization and lattice relaxation effects within their surroundings. Bipolarons can be conceptualized as a bound state involving two polarons with opposite spins, resulting in two electrons or holes on the same lattice or molecular segment.^52,53^ In the realm of charge transport, bipolarons are often disregarded in organic semiconductors due to the presumed dominance of Coulomb repulsion between the two charges forming the bipolaron, outweighing the stability gained through nuclear reorganization.^54^ However, experimental observations have revealed the presence of bipolarons in various organic devices such as organic heterojunctions, thin-film transistors, and solar cells.^55−57^ Notably, under conditions of high polaron density, the formation of bipolaron has been observed and is linked with changes in absorption spectra. We have hypothesized that the density of bipolarons is notably enhanced at the polymer/nanocluster interface, resulting in a significant increase in current leading to the formation of the LRS. To substantiate this hypothesis, we prepared a three-layer PTB7-based transmissive device and performed in situ UV–visible–NIR absorption spectroscopy of our organic memristors under applied bias, and the results are shown in Figure 5a. We have considered the three-layer configuration as a representative to explain the mechanism. With no applied bias, the distinct absorption peaks corresponding to PTB7 are clearly evident. At 1 V, the absorption patterns remain consistent. Intriguingly, at 2 V, a small absorption peak at 1072 nm emerges. Given that the *V*_C_ for the device is ∼2.3 V, the small absorption peak signifies the initiation of carrier injection at the polymer/nanocluster interface. Further increasing the applied bias to 3 V results in a pronounced absorption peak at 1100 nm, indicative of a substantial influx of bipolarons in the semiconducting layer. At this juncture, the device is fully switched on, as evidenced by the absorption peak at 1100 nm. This phenomenon becomes more prominent at 5 V as shown by a further increase in the absorption peak. Subsequently, during the reset phase (−1 V), the device’s absorption returns to the off-state pattern, closely resembling the absorption spectra observed in the unbiased configuration.

Furthermore, Figure 5b illustrates the absorption intensity at 1100 nm over time. At 0 V bias, the absorbance (∼0.063) remained constant for 30 s. With a 2 V bias, the absorbance increased to ∼0.156, signaling the onset of doping. Notably, when the applied bias is equal to *V*_C_ (2.3 V), the absorbance marginally exceeds that recorded at 2 V. Interestingly, after some time, a sudden jump in absorbance occurs, indicating the influx of bipolarons, as previously discussed. At biases >*V*_C_, the absorbance increased and remained constant throughout the time scale, representing the on-state of the device. Resetting the device (−1 V) brings the absorbance close to the unbiased condition, indicating a return to the off-state. Finally, we estimated the SCP’s band gap energy before and after the doping mechanism using the Tauc method, revealing band gap values of 1.67, 1.09, and 0.99 eV at 0, 2, and 5 V, respectively (Figure S7, Supporting Information). In Figure 5c, the band diagram illustrates the formation of polaronic and bipolaronic bands before and after applying *V*_C_. The standard band gap for PTB7 is ∼1.7 eV, and the electronic structure associated with polarons (*E*_g_ = 1.09 eV), however, can change if the holes pair at high concentration to form bipolarons (*E*_g_ = 0.99 eV). To validate this, we conducted temperature-dependent *I–V* measurements on the device within the 150–340 K range (Figure S8, Supporting Information). Employing the Arrhenius equation^58^ and analyzing the slope of ln(*I*) – 1000/*T*, we determined the activation energy to be 12.1 meV. This value is notably higher for PTB7 compared to 5–8 meV reported in the literature.^59−62^ This elevated activation energy is attributed to the motion of charge carriers within the polymer domains, particularly in the context of bipolarons. Bipolarons involve the simultaneous movement of two charge carriers, resulting in a significantly higher activation energy.

#### 2.2.3 Multilevel Switching

**Figure 6:**
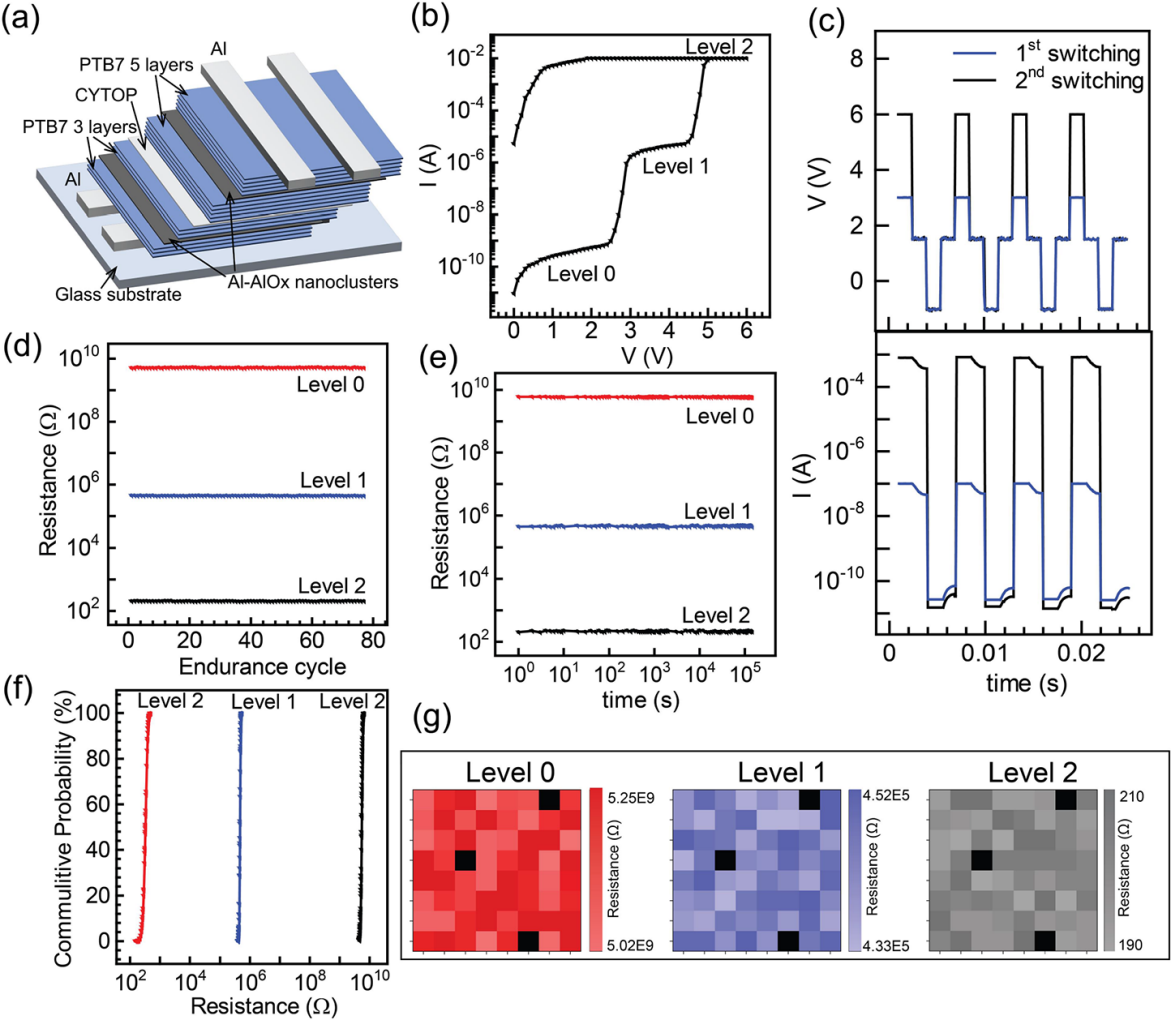
Multilevel device characteristics: (a) Schematic showing the device architecture, (b) *I–V* characteristics, (c) AC response showing *V–t* and its corresponding *I–t* characteristics, (d) Endurance cyclability of level 0 (red), 1 (blue), and 2 (black), (e) retention time of level 0, 1, and 2, (f) Cumulative probability data of level 0, 1, and 2, and (g) device-to-device uniformity for the multilevel switching-based devices.

A versatile multilevel switching device architecture characterized by the strategic stacking of two distinct and oriented SCP layers positioned one above another separated by a thin (∼10 nm) dielectric (CYTOP) layer was fabricated, as shown in Figure 6a. This innovative device architecture offers a unique approach to customize multiple switching levels within a single device. Numerous publications have explored multilevel switching using compliance current methods, where current levels are restricted to prevent device degradation.^63,64^ Nevertheless, the imposition of compliance current introduces nonidealities in switching behavior and frequently requires additional programming steps, contributing to higher power consumption. In contrast, our organic memristors, realized through the strategic stacking of different active layers with facile and independent control, present a novel and efficient approach to achieve tunable multilevel switching. The tunability of the devices is facilitated by the controlled modulation of the thickness of the SCP layers. In this work, we have fabricated the multilevel switching memristors by stacking a 3-layer configuration over a 5-layer one separated by a thin dielectric layer. This specific design was used as a representative model to exhibit the multilevel switching of the proposed device architecture. The *I–V* characteristic of the device is shown in Figure 6b. At a 0 V bias, the device resides in the off-state, exhibiting a current of 7.4 × 10^–11^ A (level 0). With a gradual increase in voltage, the current starts increasing while maintaining the off-state status until reaching the first critical voltage (*V*_C1_) at 2.7 V. At this point, the current undergoes a significant rise to 2.4 × 10^–6^ A (level 1). Further increase in voltage results in a gradual increase in current until a second critical voltage (*V*_C2_) is reached at 4.9 V, leading to another substantial increase in current to 10^–2^ A (level 2). Thus, the device architecture demonstrates multilevel switching in its *I–V* characteristic. In the previous section, we discussed that 3-layer and 5-layer configurations exhibit their individual *V*_C_ of 2.3 and 4.6 V, respectively. In the multilevel device configuration, which is composed of vertically stacked 3-layer and 5-layer configurations, it can be understood that at 0 V, both configurations are in the off-state (level 0). Upon crossing the *V*_C1_ (2.7 V), the 3-layer configuration switches on, while the 5-layer configuration remains off, resulting in an intermediate state (level 1). Subsequently, upon surpassing *V*_C2_ (4.9 V), both 3- and 5-layer configurations switch on, establishing level 2. This design enables multilevel switching by integrating different configurations in the vertical direction, significantly increasing the device density. Notably, the advantage of this configuration lies in its ease of fabrication and independence from current compliance requirements for achieving multilevel switching. The devices showcase a remarkable *I*_on (level 1)_/*I*_off (level 0)_ of 4 × 10^4^ and *I*_on (level 2)_/*I*_off (level 0)_ of 1.3 × 10^8^. These results obtained by multilevel switching clearly demonstrate the prospects of increasing the switching levels by the simple and reliable fabrication approach and incorporation of additional layer configurations.

Next, we examined the AC characteristics of the device, and the results are shown in Figure 6c. The writing process occurred at a voltage marginally exceeding *V*_C1_ (3.2 V) for the first switching (level 1) and *V*_C2_ (5 V) for the second switching (level 2). During a subsequent reading at 1 V, the device maintained the level 1 state and level 2, respectively, indicating nonvolatile memory behavior. Later at −1 V, a succeeding transition from on to off-state (level 0) is observed. We quantified the switching speed of the devices by assessing their AC characteristics (Figure S9, Supporting Information). Our analysis revealed that even after the incorporation of multilevel switching, the devices retained their switching speed (∼90 ns). The on-and-off endurance cyclability of the device was systematically tested, revealing stability for 87 cycles with <1% deviation observed throughout the cycling process, as shown in Figure 6d. Upon investigating the long-term retention times of the HRS and LRS (Figure 6e), we observed no obvious decline over an extended duration of 2 × 10^5^ s. To show the device-to-device variation, we fabricated 64 devices on a 2 cm × 2 cm substrate, each with an area of 6.25 × 10^–2^ mm^2^ as illustrated previously. Figure 6f exhibits a distribution of the resistance for 64 devices. The mean resistances for levels 0, 1, and 2, were 5.17 × 10^9^ ± 1.81 × 10^8^, 4.45 × 10^5^ ± 3.42 × 10^4^, and 301.8 ± 18.4 Ω, respectively. The distribution of set voltages for the 64 devices is shown in Figure S10, Supporting Information, showcasing each distinct level with no interference. The incorporation of different layer configurations in 3D doubled the density of each device with minimal crosstalk. This device architecture was facilitated by the straightforward LbL fabrication using UFTM. The high uniformity of the devices and the capability of fabricating high-density devices in a small area, with minimal cross-talk is also demonstrated, as shown in Figure 6g. Notably, >95% of devices exhibited uniform functionality. The I–V characteristics of all the devices are shown in Figure S11, Supporting Information. The consistent performance witnessed in the devices can be attributed to the exceptional uniformity achieved by UFTM films. Additionally, device-to-device variation for 30 different batches is presented in Figure S12, Supporting Figure. Here, we considered the average on–off ratio for 64 devices in each batch. There is a variation of less than 5% in the different batches. The high uniformity is maintained in the LbL deposition, making this method suitable for high-performance vertical devices, thus signifying the excellent reliability and stability of our memristor.

#### 2.2.4 Flexible Multilevel Memristors

**Figure 7:**
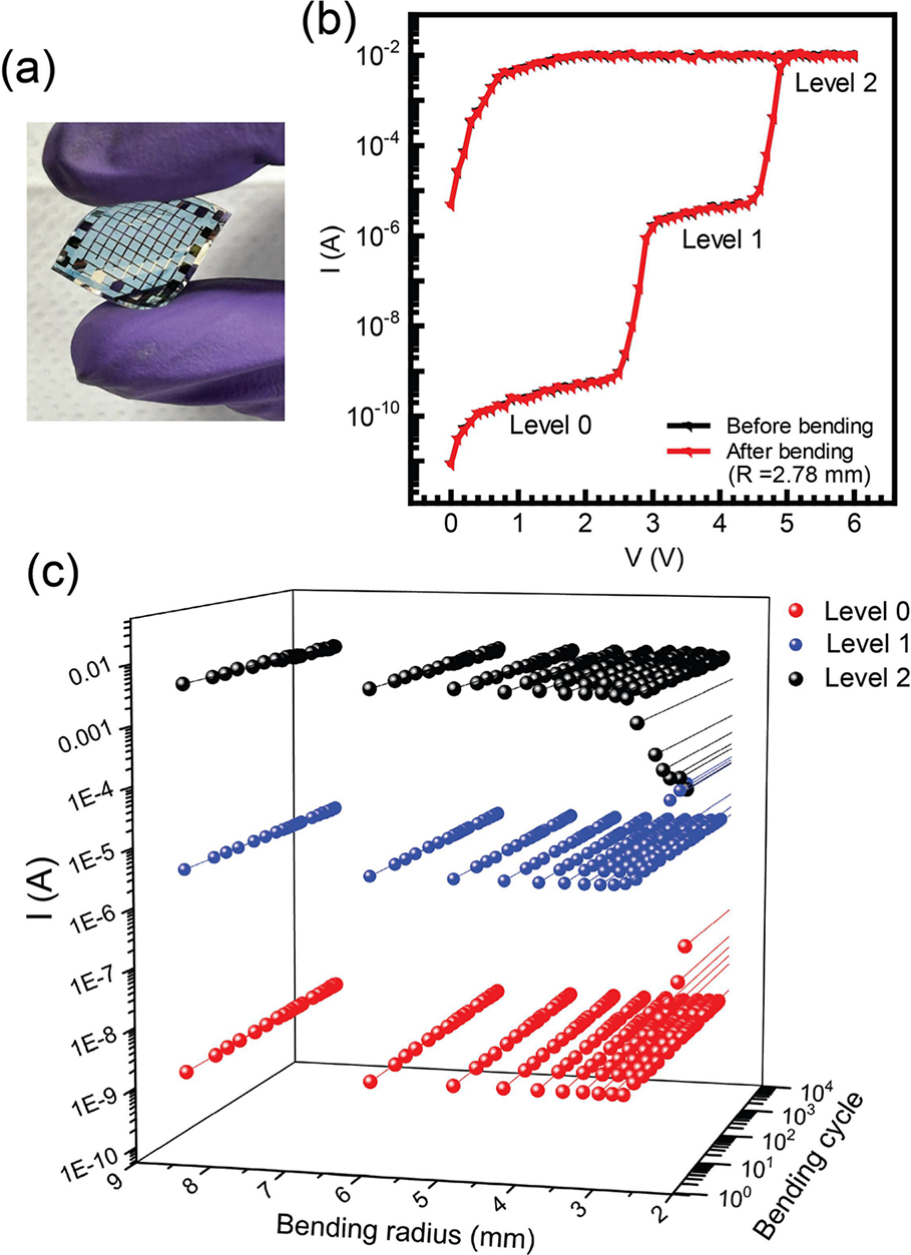
(a) Picture of the flexible memristor. (b) *I–V* characteristics before (black) and after (red) bending the flexible multilevel memristors. (c) Current evolution with bending radius and bending cycles showing the three levels.

As flexible memory devices, organic memristors exhibit promising potential, showcasing enhanced adaptability and functionality within electronic systems.^65,66^ In the study, we successfully fabricated flexible devices, highlighting the sustained performance of our multilevel memristor devices on flexible substrates. Devices were fabricated on a 100 μm thick PET substrate, as shown in Figure 7a. The entire device had a very small thickness (≈500 nm) in comparison to the substrate. The *I–V* characteristics were measured both before and after bending at a radius of 2.78 mm as shown in Figure 7b. It is clear that there is no discernible deviation in the *I–V* characteristics of the device after bending. The bending radii (*R*) of our flexible devices can be described by the sinusoidal curve^67^ as
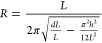
6where *h*, *L*, and *dL* are the thickness of the sheet, the length of the device, and the change in the length along the bending direction, respectively.

The *I–V* characteristics were also measured for multiple bending cycles conducted for devices (≥10^4^ cycles), and the results are shown in Figure 7c. Notably, the devices retained almost the same current values at a bending radius of 2.78 mm for ≥10^4^ cycles. Despite the low bending radii, these devices exhibited commendable performance. We also evaluated the stress–strain curve of our flexible devices (Supplementary Figure S13). It can be seen that the device stress exerted by our flexible device was linear with respect to the strain, indicating its ability to undergo reversible deformation and return to its original shape. This observation supports the consistency in the device’s *I–V* characteristic. Young’s Modulus of the devices was determined to be 0.26 GPa, a typically low value suitable for flexible device applications.^68,69^ Finally, Table 1 presents the recent trends in organic resistive memory devices, offering a comparative overview that includes the positioning of our devices within this context.

**Table 1:** Comparison Table of Recent Organic Resistive Memory Devices Reported Previously

device structure	on–off ratio	retention time (s)	endurance cycles	multilevel	flexibility	operating voltage (V)	reference
Al/PTB7/Al-AlO_*x*_/PTB7/Al	10^8^	10^5^	∼100	yes	yes	3.2, 5	this work
Cu/graphene/PMMA:P3BT/Al	10^3^	10^4^		no	yes	4.1	(70)
ITO/pEGDMA/Cu	10^2^	10^6^	10^3^	no	yes	1	(71)
Au/EGC-1700–3M/Ag	10^5^	10^4^	50	yes	yes	3.5	(72)
Pt/AlOOH/ITO	10^2^	10^3^	500	yes	no	3,1	(73)
Multilayer graphene/PI:PCBM/Al	10^6^	10^4^	10^4^	no	yes	4.2	(74)
ITO/Parylene/Cu	10^5^	10^2^	10^3^	yes	no	5	(75)
Cu/graphene/PMMA:P3HT/Al	10^5^	10^3^	80	no	yes	3.6	(76)
ITO/BCPO/Al	10^4^	10^4^	-	no	no	4	(77)
ITO/[Ru(L)3](PF6)2/Au	10^5^	10^12^	10^12^	no	no	4.2	(78)
ITO/PQT/PEO + EV(ClO4)2/Au	10^5^	10^4^	10^3^	no	no	3–5	(79)
Pt/PTEDOT-AuNP/Pt	10^3^	10^5^	800	no	no	5	(80)
Al-AlO_*x*_/OTS/Al-AlO_*x*_/OTS/Pentacene/Au	10^5^		10^3^	no	yes	6	(81)
ITO/PEDOT:PSS/CS-rGO/Ag	10^3^	10^4^	100	yes	yes	1	(82)

## 3 Conclusions

In summary, this work presents the successful fabrication and comprehensive characterization of organic memristors using the SCP PTB7. Employing UFTM and LbL, a memory device in a crossbar architecture with Al/PTB7/Al-AlO_*x*_-nanoclusters/PTB7/Al was successfully fabricated. Systematically varying the number of layers of PTB7 films in the devices revealed a correlation between the thickness of the semiconducting layer and the resistance of LRS and HRS. Concurrently, the *V*_C_ of the devices shifted toward the positive bias. The devices demonstrated exceptional performance metrics including a high on–off ratio (>10^5^), excellent retention (>10^5^ s), robust endurance (∼100 on–off cycles), and fast switching speed (∼100 ns). An array of devices fabricated on a 2 cm × 2 cm substrate (64 crossbar devices; device size = 6.25 × 10^–2^ mm^2^) demonstrated an efficacy of >95% with minimal crosstalk. We elucidated the charge transport mechanism, attributing it to the presence of Al/AlO_*x*_ nanoclusters with the charge transport process explained using the Hubbard model. The switching-on (LRS) of our memristors was attributed to the influx of a huge concentration of bipolarons, leading to a sharp increase in the current after the applied bias exceeded *V*_C_. We also proposed and implemented a novel vertically stacked device crossbar architecture for multilevel switching for the device. Here, we fabricated devices with three-layer and five-layer configurations separated by a 10 nm dielectric layer, exhibiting three levels, effectively doubling the device density. These multilevel memristors demonstrated a high on–off ratio (>10^8^), remarkable memory retention (>10^5^ s), high endurance (87 on–off cycles), and rapid switching (100 ns). We have also shown the implementation of these multilevel memristors on a flexible substrate, where consistent performance was retained even after bending at a radius of 2.78 mm for 10^4^ cycles. This study not only enhances the fundamental understanding of charge transport mechanisms in organic memristors but also presents avenues for advanced device architectures for multilevel memory and flexible devices.

## 4 Methods

### 4.1 Materials

Electronic-grade PTB7 was purchased from 1-Molecule and used without further purification. Ethylene glycol, superdehydrated chloroform (>99.99%), hexane, acetone, methanol, and isopropyl alcohol (IPA) were purchased from Sigma-Aldrich.

### 4.2 Thin Film Fabrication

PTB7 ink was prepared in superdehydrated chloroform (2% w/v), and ∼10 μL of ink was dropped at the tip of a customized slider over a pool of hydrophilic liquid substrate ethylene glycol at room temperature (Figure 1a, Supporting Video 1). The SCP ink spreads over the liquid substrate due to the Marangoni Effect. The customized slider restricts the flow of the SCP ink in a single direction. As the ink spreads, the solvent (chloroform) evaporates, thus resulting in the formation of a thin floating SCP film of 15 × 2 cm over the liquid substrate. The floating films can be cast over any desired solid substrate (rigid/flexible). Later, any residual ethylene glycol was washed away gently using methanol and dried using nitrogen blow, and the process was repeated for LbL coating to increase the film thickness or fabricating a multilevel memory device over a thin dielectric layer. The PTB7 films were annealed on a preheated hot plate (100 °C for 30 min) in an argon glovebox once the desired thickness was completed by LbL coating. Finally, the substrates were left to slowly cool down to room temperature.

### 4.3 Device Fabrication and Characterization

The glass, PET, and Si substrates were first cleaned using hexane and sonicated in acetone, IPA, and chloroform for 10 min each. The substrates were then annealed at 100 °C for 1 h. Later, the Al bottom layer (50 nm) was thermally evaporated under a high vacuum (∼10^–6^ Torr) at a rate of 1 Å s^–1^ using a 250 μm thick mask. Over this, 3, 4, and 5 layers of floating films were deposited as described previously. After this, an 8 nm layer of Al/AlO_*x*_ nanoclusters was thermally evaporated in a 10^–6^ Torr vacuum. For this, Al was thermally evaporated under ∼10^–6^ Torr at a very slow evaporation rate (<0.1 Å s^–1^). The presence of residual oxygen in the evaporation chamber initiates the oxidation of a portion of the deposited aluminum with the subsequent deposition of unoxidized aluminum. This process leads to the formation of distinct layers comprising AlO_*x*_ nanoparticles interspersed within layers of Al nanoparticles. Next, 3, 4, and 5 layers of floating films were again deposited, followed by thermal deposition of the top Al contact (50 nm) as mentioned previously. For multilevel switching, over the floating films, a 10 nm CYTOP film was deposited by spin-coating diluted CYTOP (CYTOP:CT solv. = 1:30) at 3000 rpm for 60 s followed by annealing at 100 °C for 1 h. Then again, another five-layer configuration was deposited in the same LbL manner, followed by the deposition of top Al contact (50 nm). *I–V* characteristics were measured with a computer controller two-channel source meter (Keithley 5612).

### 4.4 Thin Film Characterization

Si substrates were used to prepare for X-ray characterization. The films were characterized using out-of-plane X-ray diffraction (θ–2θ scan) on a Rigaku X-ray diffractometer with a Cu–K radiation source. Incident X-rays with a refractive index <1 undergo total external reflection when their grazing incidence angle (ω) with the film surface is < the critical angle (ω_c_). For in-plane GIXD (φ–2θχ scan), the sample and detector underwent rotations by angles of φ and 2θχ, respectively, while the scattering angles were set to 0.14 and 0.28° from the sample surface, respectively. For out-of-plane XRD measurements, the X-ray source and detector underwent rotations by angles of θ and 2θ, respectively, from the specimen plane. The electronic absorption spectra of the devices were obtained using Ulvac V-570. We fabricated a memristor that was transmissive but exhibited similar device performance as discussed in the manuscript. For this, first, 10 nm Al was deposited, followed by 3 layers of PTB7 floating films. Then, another 6 nm Al/AlO_*x*_-nanoclusters were deposited as discussed above, with subsequent deposition of three layers of PTB7 floating films. Finally, a 10 nm Al top contact was deposited. Bias was applied to the top and bottom contacts of the device, and an in situ absorption spectra were measured. Background absorbance measurements of the device were taken without PTB7 films and then subtracted from the original results.

## Supporting Information Available

Detailed device characteristics for bistable switching, multilevel switching, and flexible organic memristors and details on device mechanism (PDF)
Explanatory video of UFTM (MOV)

## Supplementary Material

am4c03111_si_001.pdf

am4c03111_si_002.mov
