# Supplementary material for: High-Density, Nonvolatile, Flexible Multilevel Organic Memristor Using Multilayered Polymer Semiconductors
Source: ACS Appl Mater Interfaces. 2024 Apr 22;16(17):22282–93. doi: 10.1021/acsami.4c03111 (PMC11082853; doi:10.1021/acsami.4c03111)
Supplement: Supplementary file 1 — am4c03111_si_001.pdf [file am4c03111_si_001.pdf]

Supporting Information

High-density, non-volatile, flexible multilevel organic memristor using multilayered polymer semiconductors

Shubham Sharma[1], Manish Pandey[2], Shuichi Nagamatsu[3], Hirofumi Tanaka[4], Kazuto Takashima[1], Masakazu Nakamura[5], Shyam S. Pandey[1*]*

[1]Graduate School of Life Science and Systems Engineering, Kyushu Institute of Technology, 2-4 Hibikino, Wakamatsu, Kitakyushu 808-0196, Japan.

[2]Department of Electronics and Communication Engineering, Indian Institute of Technology, Bhilai, Durg, Chattisgarh, India, 491001

[3]Department of Computer Science and Electronics, Kyushu Institute of Technology, 680-4 Kawazu, Iizuka, 820-8502, Japan.

[4]Department of Human Intelligence Systems, Kyushu Institute of Technology, 2-4 Hibikino, Wakamatsu, Kitakyushu 808-0196, Japan.

[5]Division of Materials Science, Nara Institute of Science and Technology, Ikoma, Nara 630-0192, Japan.

[*]Corresponding authors: S.S.: shubhammudgal95@gmail.com; S.S.P.: shyam@life.kyutech.ac.jp

Keywords: semiconducting polymers, resistive memory devices, floating film transfer method, multilevel switching, flexible devices.

TABLE OF CONTENTS

Figure S1: Thickness of 3, 4, 5 layers of UFTM films

Figure S2: Mean values of critical voltage (V_C)

Figure S3: Switching speed for 3. 4. And 5 layered devices

Figure S4: Cumulative probability for 3, 4, and 5 layered devices

Figure S5: I – V characteristics for all 64 3, 4, and 5 layered devices

Figure S6: Device-to-device variation for 3, 4, and 5 layered devices

Figure S7: Tauc plot of organic memristors at 0, 2, and 5 V

Figure S8: Temperature dependent I – V characteristics and Arrhenius plot for organic memristors

Figure S9: Switching speed for multilevel organic memristors

Figure S10: Cumulative probability for multilevel organic memristors

Figure S11: I – V characteristics for all 64 multilevel organic memristors

Figure S12: Device-to-device variation for multilevel organic memristors

Figure S13: Stress – strain characteristics for flexible organic memristors

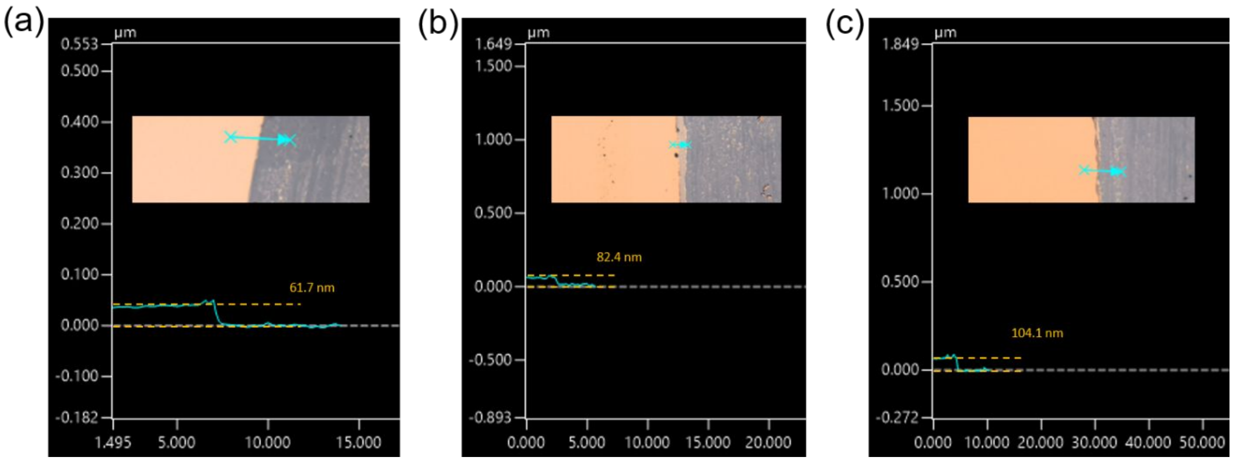

Supporting Figure S1: Thicknesses of (a) 3 layers, (b) 4 layers, and (c) 5 layers of UFTM thin films measured using Keyence 3D surface profiler. It can be attributed from the figure that the average thicknesses of 3, 4, and 5 layers of UFTM films are 61.7 nm, 82.4 nm, and 104.1 nm, respectively. This gives the average thickness of one film of approximately 20 nm.

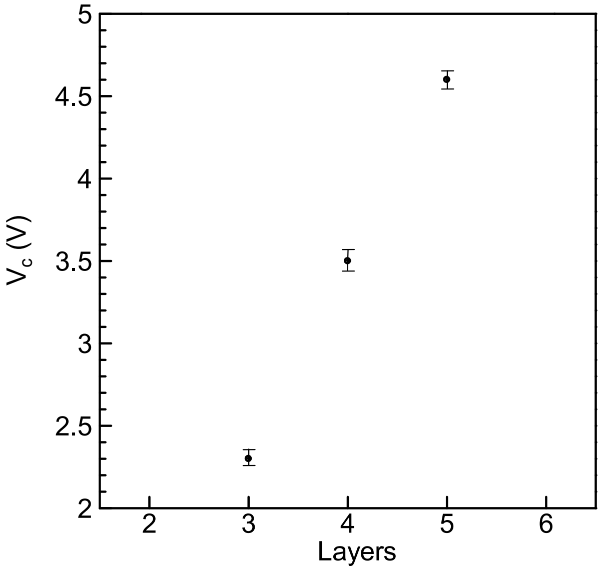

Supporting Figure S2: The mean values of the critical voltages (Vc) with error bar for 3, 4, and 5 layered film based organic memristors. It can be seen that the margin of error in the values of Vc is minimal and the critical voltage increases linearly with the increase in number of layers in the

device. This is vital because on further changing the thickness of the semiconductor films, we can predict the critical voltage which can further help us in controlling the multilevel switching.

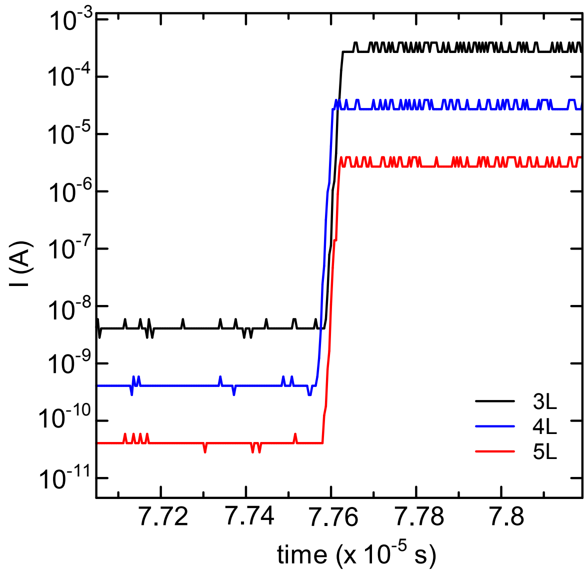

Supporting Figure S3: AC characteristics with current vs time for 3, 4, and 5-layer devices showing the switching speed of the devices. It can be attributed that the swathing speed is 98.1, 97.3, and 99.8 ns for 3, 4, and 5 layer devices, respectively.

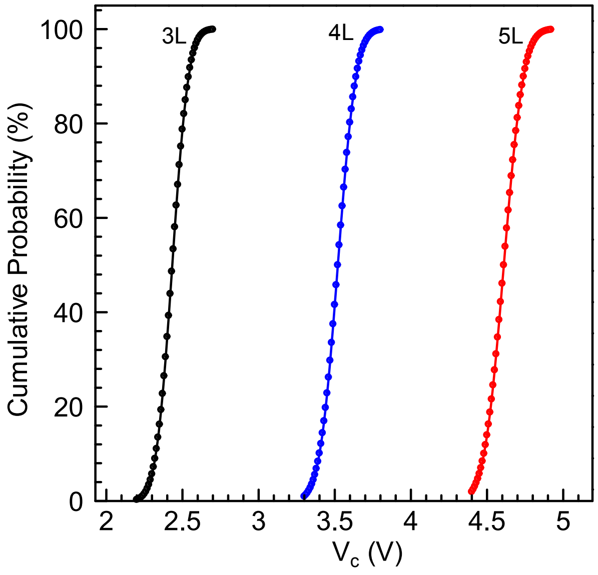

Supporting Figure S4: Cumulative probability for 3, 4, and 5-layer devices exhibiting the mean values of V_c to be 2.3 ± 0.8 V, 3.5 ± 0.7 V, and 4.6 ± 0.7 V, respectively.

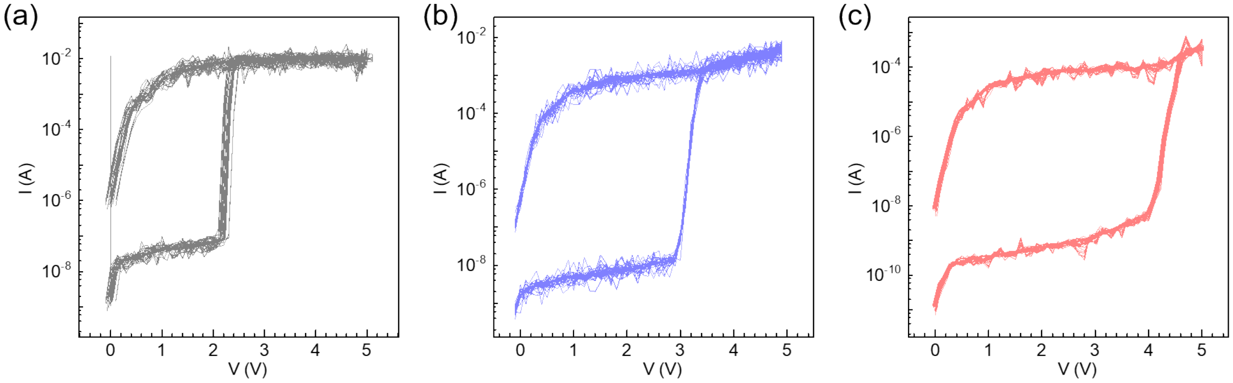

Supporting Figure S5: I-V characteristics of all the 64 (a) 3 layer, (b) 4 layer, and (c) 5 layer-devices exhibiting minimal deviation.

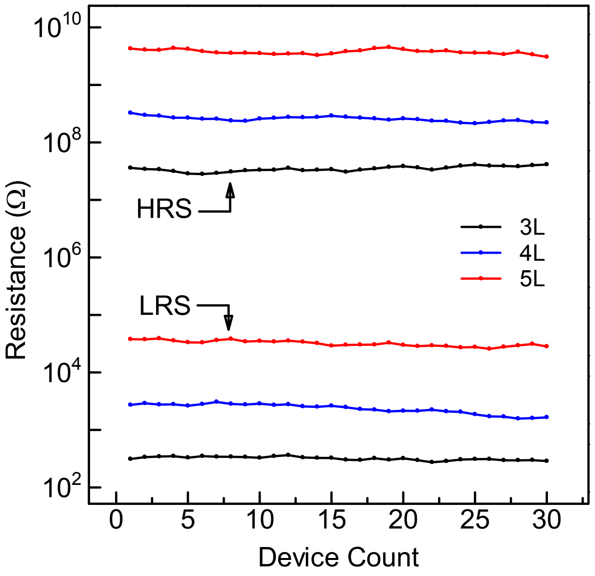

Supporting Figure S6: Device-to device variation for 3 layers (3L), 4 layers (4L) and 5 layers (5L)

The average LRS and HRS values for the 64 cells on each device substrate was calculated to find the device-to-device variation in our organic memristors. Here, <5% error exists in the resistance values.

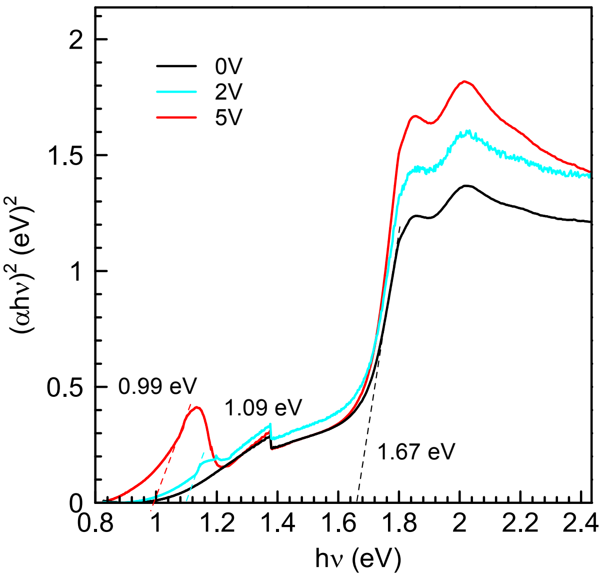

Supporting Figure S7: Tauc plot for the organic memristors at a bias of 0, 2, and 5V.

The Tauc method is a well-established approach for determining the bandgap energy of a material by analyzing its absorption spectrum. In this method, the square of the absorption coefficient (α) multiplied by the photon energy (hν) is plotted against photon energy (hν). the Tauc plot exhibits a linear region at lower photon energies. Identify this linear portion of the plot, as it is essential for determining the bandgap. By extending the linear portion of the plot intersecting the x-axis gives an estimate of the bandgap energy (Eg).

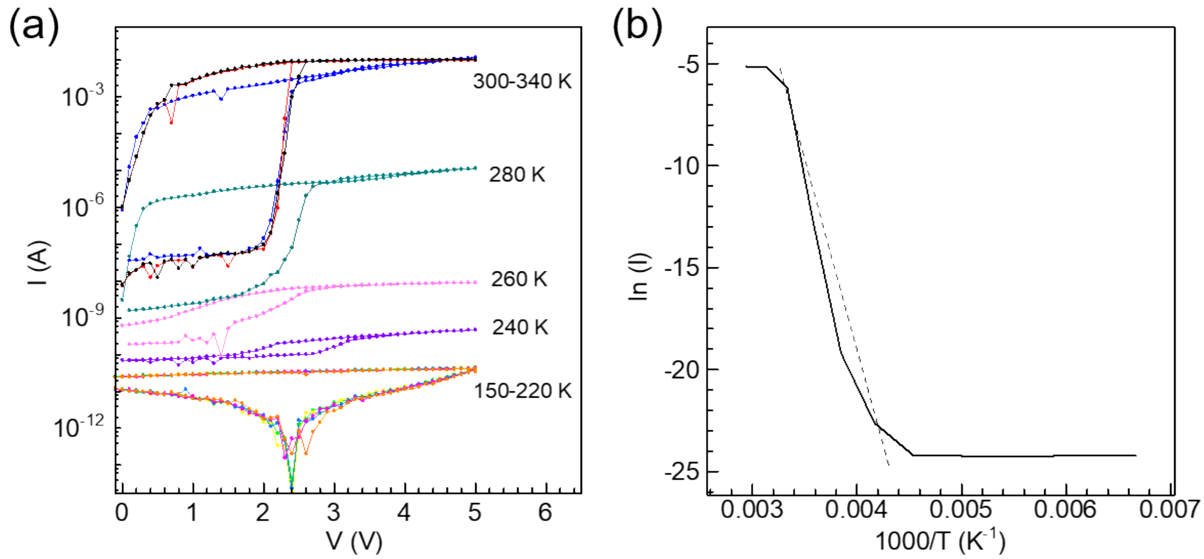

Supporting Figure S8: (a) Temperature dependent I-V characteristic, and (b) Arrhenius plot showing ln(I) vs 1000/T curve for the 3-layer organic memristors.

Using the Arrhenius equation

$$I = I_0 e^{-\frac{E_a}{kT}}$$

where I is the current, I_0 is the pre-exponential factor, E_a is the activation energy, k is the Boltzmann constant, and T is the temperature.

From the slope of ln(I) vs 1000/T, Ea was calculated to be 12.1 meV. The slope was evaluated from the linear region of the curve and not from higher or lower temperature regions.

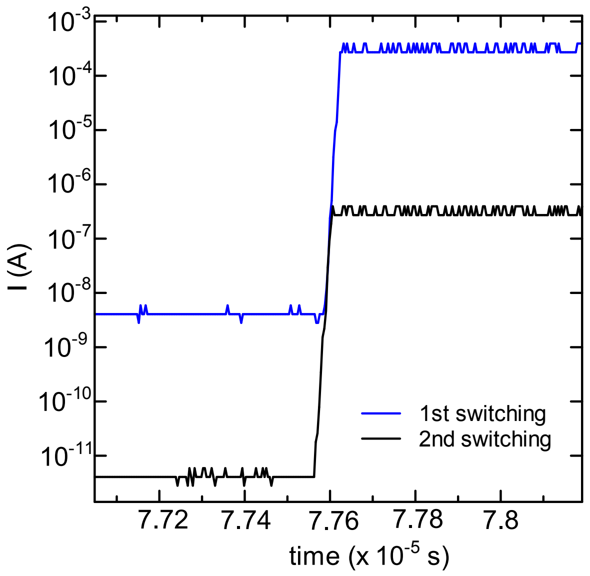

Supporting Figure S9: AC characteristics with current vs time for 1st and 2nd switching for the multilevel devices showing the switching speed of the devices. It can be attributed that the switching speed is 95.4, and 92.5 ns for 1st and 2nd switching, respectively.

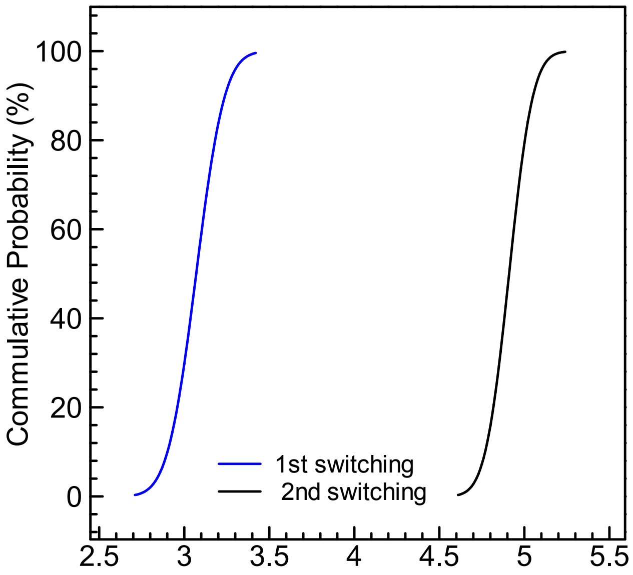

Supporting Figure S10: Cumulative probability for the 1st and 2nd switching for the multilevel devices with the mean values of V_c to be 3.2 ± 0.5 V, and 5 ± 0.6 V, respectively.

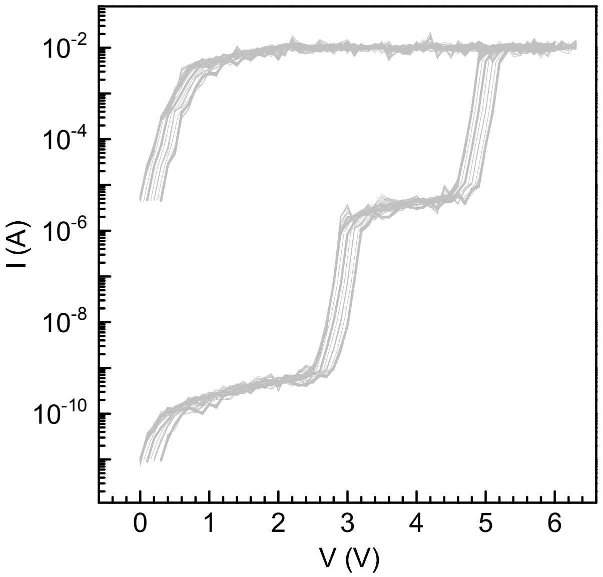

Supporting Figure S11: I-V characteristics of all the 64 multilevel devices.

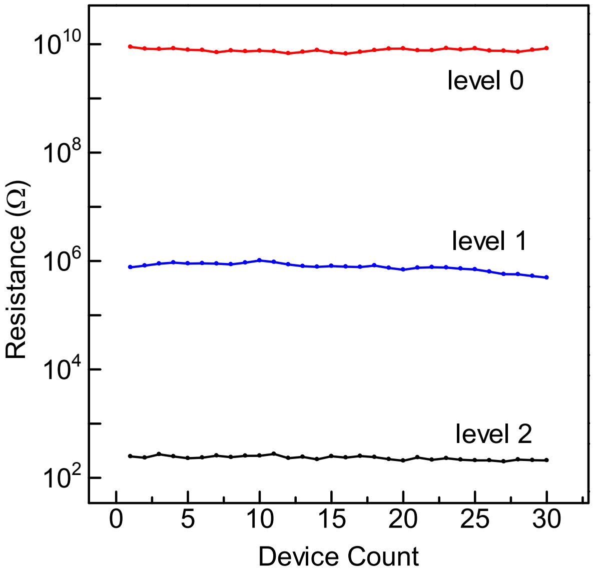

Supporting Figure S12: Device-to device variation for multilevel device

The average LRS and HRS values for the 64 cells on each device substrate was calculated to find the device-to-device variation in our organic memristors. Here, <5% error exists in the resistance values.

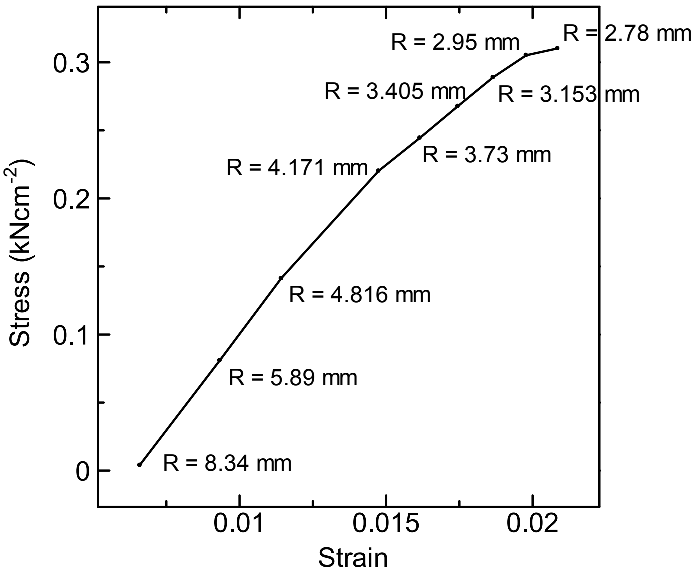

Supporting Figure S13: Stress vs Strain curve for the flexible organic memristors. The slope of the linear region of the curve gives the Young's Modulus. (R = bending radius)
